# Potent *In Vitro α*-Glucosidase and *β*-Secretase Inhibition of Amyrin-Type Triterpenoid Isolated from *Datura metel* Linnaeus (Angel's Trumpet) Fruits

**DOI:** 10.1155/2020/8530165

**Published:** 2020-08-25

**Authors:** Saud Bawazeer, Abdur Rauf, Sami Bawazeer

**Affiliations:** ^1^Department of Pharmaceutical Chemistry, Faculty of Pharmacy, Umm Al-Qura University, Makkah, P.O. Box 42, Saudi Arabia; ^2^Department of Chemistry, University of Swabi, Swabi-Anbar-, 23430 KPK, Pakistan; ^3^Department of Pharmacognosy, Faculty of Pharmacy, Umm Al-Qura University, Makkah, P.O. Box 42, Saudi Arabia

## Abstract

This study deals with *α*-glucosidase and *β*-secretase inhibitory screening of extract/fractions and isolated daturaolone (**1**), namely, 3-oxo-6-*β*-hydroxy-*β*-amyrin (daturaolone) from chloroform fraction of *Datura metel* L. Among entire fractions, the chloroform soluble fraction showed excellent activity against *α*-glucosidase with % inhibition 90.8 with IC_50_160.2 ± 1.85 *μ*g and daturaolone (**1**) with 98.7% inhibition with IC_50_840.4 ± 1.74 *μ*M, respectively. Similarly, extract and daturaolone (**1**) also exhibited significant activity against the *β*-secretase enzyme (BACE1) with % activities 88.27 and 95.19 and with IC_50_ values 304.21 ± 2.98 *μ*g and 260.70 ± 1.87 *μ*M, respectively, as compared to the standard inhibitor (Ans670, Sta671, Val672)-amyloid-*β*/A4 precursor protein 770 fragments 662-675) with % activity 94.21 and IC_50_ value 289.24 ± 1.60 *μ*M. This finding encourages and opens a new window for further detail phytochemical investigation on *D*. *metel* in order to isolate novel compounds with promising enzyme inhibitory potential.

## 1. Introduction

*Datura metel* belongs to family Solanaceae which grows throughout the year and is normally known as devil's trumpet [1, 2]. *D*. *metel* is nourished in the humid and hot climate. It is found in India, Pakistan, Africa, and South America [3]. Its flower is long and possessing a white color and has a purple color that scented up to 6 inches [4], while the leaves are green in color and bread in shape. *D*. *metel* plant length ranges from 10 to 20 cm and broad from 5 to 18 cm. Its fruits are 4-10 cm thick and like a spiny capsule. *D*. *metel* is a rich source of manufacturing numerous secondary metabolites such as saponins, flavonoids, triterpenoids, alkaloids, steroids, and tannins [4]. The biological potency of *D*. *metel* is correlated due to the presence of secondary metabolites. An alkaloid known as scopolamine is a major compound isolated from *D*. *metel* has been reported for the treatment of different diseases such as bronchitis and asthma [2]. *D*. *metel* is also used for the cure of epilepsy, heart disease, fever, diabetes, insanity, diarrhea, and a skin disease [5, 6]. It is also a key source of producing anolides, which is also used for the treatment of pain and also have hallucinogenic strength [7–9]. Its seeds are used for the treatment of dental pain. It has extensive application in ayurvedic medication such as many classes of compounds isolated from the title plant that is used to treat hair fall and many skin-related infection disease [10]. The plant extracts of *D*. *metel* has been reported for anti-inflammatory and antimicrobial effects [11, 12]. Phytochemical *D*. *metel* is discovered very slightly; atropine reported from *D*. *metel* is used to dilate the pupil and it helps in the surgery of eyes [13]. The *α*-glycosidase catalyzed the hydrolysis of glycosidic bonds in glycoconjugates as well as polysaccharides and act an acute role in different biological processes, such as lysosomal catabolism of glycoconjugates, posttranslational changes of cellular glycoproteins, and digestion of carbohydrate [14, 15]. Particularly, human *α*-glycosidase enzymes in the mucosal brush edge of the small intestine catalyzed the finale step of digestion of disaccharides and starch present in human food. *α*-Glycosidase in the small intestine is selectively involved in the hydrolysis of terminal (1→4) connected *α*-glucose residues (disaccharides/starch) to release a single *α*-glucose molecule [14]. Potent *α*-glycosidase inhibitor delay is involved in the reeducation of the postprandial blood glucose excursion and decomposition of carbohydrates in the small intestine; therefore, the inhibitor of *α*-glycosidase has an important effect on metabolism polysaccharide, cellular interaction, glycoprotein processing, and wide chances for the development of new and novel therapeutic agents against various diseases including cancer, viral infection, and metastatic diabetes [15–17]. *β*-Secretase is an aspartic proteinase that is involved in numerous functions such as physiological processes in humans, including immunoregulation, death of the cell, and cell differentiation [18]. There are several pieces of evidence that a change in *β*-secretase action is involved in various diseases including schizophrenia, epileptic behavior, and Alzheimer's diseases [19]. *β*-Secretase enzyme is mainly involved in the breakdown of *β*-amyloid precursor protein at one of the two decompositions required to produce *β*-amyloid peptides. *β*-amyloid peptides are the main compound of amyloid plaques in Alzheimer's disease brain and act as a key role in the pathogenesis of divesting neurodegenerative disorder [18]. The finding deals with the *α*-glucosidase and *β*-secretase inhibitory screening of extract/fractions and isolated daturaolone (**1**), namely, 3-oxo-6-*β*-hydroxy-*β*-amyrin from chloroform fraction of *Datura metel* L.

## 2. Experimental

### 2.1. Plant Material Collection

The plant materials (fruits) of *Datura metel* were obtained from the hilly area of Razagram Toormang Dara, Dist; Dir, KPK, Pakistan. The plant sample was identified by Dr. Muhammad Ilyas, Head Department of Botany, University of Swabi, KPK, Pakistan. The voucher specimen no. Bot (UOS-521) was placed in the herbarium of the Botany Department of University of Swabi.

### 2.2. Extractions, Fractionation, and Isolation

**Figure 1:**
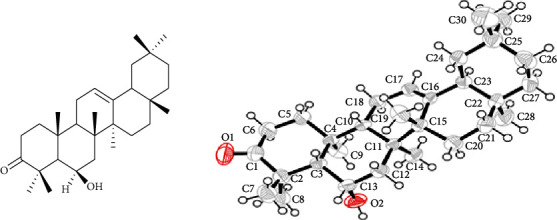
Chemical structure and X-ray crystallographic image of daturaolone (**1**) isolated from *D*. *metel.*

The shade-dried fruits (8.2 kg) were grinded with the help of a local grinder machine to obtain powder, and then, 8.00 kg powder plant material was soaked in methanol (40 liters) for 13 days to obtain a crude methanolic extract 350 g. The crude extract was suspended in water (500 mL) to prepare the paste and then successively extracted with various organic solvent on polarity, to obtain *n*-hexane extract (50.9 g), chloroform extract (93.00 g), and ethyl acetate extract (45.87 g) as per standard methods [20]. A small portion of all obtained extracts was subjected to thin-layer chromatography (TLC), by using different solvent systems; the maximum separation was observed in *n*-hexane and ethyl acetate. Based on the TLC profile, the chloroform fraction was selected for separation because it contains a maximum number of compounds. Among the chloroform extracts, 25.0 g was chromatographed on silica gel by using normal phase column chromatography. The column was eluted with a mixture of *n*-hexane and ethyl acetate (0 : 100) of increasing polarity order. Two hundred fractions were collected, which were combined to 16 major fractions (SB-1-SB16), as per the TLC profile. Based on TLC profile, SB-9 was subjected to repeated pencil column chromatography (CC), eluting with *n*-hexane and ethyl acetate (14 : 86), which yielded white crystal. The obtained crystals were washed with *n*-hexane which gives daturaolone (**1**; 99.89% pure) (2.7 g). The chemical structure of daturaolone (**1)** was elucidated by advanced spectroscopic technique (^1^H-NMR, ^13^C-NMR, HMBC, HMQC, NOESY, COSY, and EI-MS). The structure was confirmed by crystallography technique (Figure 1) and compared the spectral data with the reported one [21, 22].

## 3. Enzyme Inhibitory Screening

### 3.1. *α*-Glucosidase Inhibitory Assay

The crude extracts and isolated daturaolone (**1**) were assessed for *α*-glucosidase inhibitory activity as per reported methods [23, 24]. Rat intestinal **(**CH_3_)_2_CO (acetone) powder in normal saline (100 : 1 *w*/*v*) was sonicated properly, and the supernatant was utilized as a wellspring of basic intestinal *α*-glucosidase after centrifugation. Shortly, 10 mL of tested samples (5 mg/mL) in dimethyl sulfoxide solution was reconstructed in 100 mL of 100 mM phosphate buffer at pH 6.8 out of a 96-well microplate and hatched with 50 mL of important intestinal *α*-glucosidase for 5 min before 50 mL substrate (5 mM, p-nitrophenyl-a-D-glucopyranoside agreed in the alike buffer) was included. The p-nitrophenol free was slow at 405 nm spectrophotometrically (SpectraMax plus384, Molecular Devices Corporation, Sunnyvale, CA, USA) 5 min after brooding with the substrate. Singular spaces for the test samples were fixed up to exact foundation absorbance where the substrate was altered with 50 mL of buffer. The control sample controlled 10 mL DMSO alongside test samples. The percent enzyme inhibition was calculated as

(1) $$\begin{array}{c} \left(1-B/A\right)\times 100, \end{array}$$

where *A* was the absorbance of control limited of test samples and *B* was the absorbance in the presence of test samples.

### 3.2. Beta Secretase FRET Assay

The crude extracts and isolated daturaolone (**1**) were assessed for *β*-secretase inhibitory potential as per standard procedure [25]. 82.5 *μ*L buffer contains 50 mM sodium acetate buffer with pH 4.5 was mixed to each inhibitor, in all wells, and after that, 2.5 *μ*L from 20 U/100 *μ*L solution of the enzyme (0.5 U) should be combined. The reaction mixture was incubated for 20 minutes at 25°C. Then, the reaction will be starting by combing 62.5 nM from 12500 nM/1 mL solutions of a substrate; the incubation should be again obtained for 60 minutes at 37°C in the dark condition. After incubation of a 96-well black plate, 1000 nM from 100 *μ*M/1 mL stock solution of extract/fractions and isolated daturaolone (**1**) were combined; then, the incubation time of the plate should be read by the fluorometer machine (CHAMELEON-HIDEX) as per reported standard procedure [25] . Emissions, as well as excitation for Mca, are 325 and 400 nm, respectively.

## 4. Results and Discussion

Daturaolone (**1**) was isolated from chloroform fraction and identified as 3-oxo-6*β*-hydroxy-*β*-amyrin by different spectroscopic techniques such as ^1^H-NMR, ^13^C-NMR, IR, and mass spectral data.

Daturaolone (**1**) was white crystals; IR (KBr, cm^−1^) *ν*_max_ = 1599 (C=C), 1699 (C=O), 2918 (C-H), 3650 (OH). ^1^H NMR (500 MHz, CDCl_3_): *δ* 0.83 (s, 3H, CH_3_-28), 0.85 (*s*, 6H, CH_3_-29, CH_3_-30), 1.08 (*s*, 3H, CH_3_-27), 1.15 (*s*, 3H, CH_3_-23), 1.22 (*t*, 2H, CH_2_-22), 1.32 (*s*, 3H, CH_3_-26), 1.40 (*s*, 3H, CH_3_-24), 1.49 (*s*, 3H, CH_3_-25), 1.53 (*s*, 2H, CH_2_-7), 1.62 (*t*, 2H, CH_2_-15), 1.64 (*d*, 1H, CH-5), 1.65 (*d*, 2H, CH_2_-19), 1.67 (*t*, 1H, CH-18), 1.76 (*m*, 2H, CH_2_-11), 1.98 (*t*, 2H, CH_2_-16), 2.06 (*t*, 1H, CH-5), 2.22 (3, 2H, CH_2_-1), 2.73 (3, 2H, CH_2_-2), 4.49 (brs, 1H, CH-6), 5.24 (*s*, 1H, CH_2_-12); ^13^C NMR (125 Hz, CDCl_3_): *δ* 16.5 (CH_3_-19), (CH_3_-19), 18.6 (CH_3_-26), 23.6 (CH_3_-11, 24), 23.9 (CH_3_-30), 25.8 (CH_3_-23), 25.9 (CH_3_-27), 26.1 (CH_2_-15), 26.9 (CH_2_-16), 28.3 (CH_3_-28), 31.0 (C-20), 32.8 (C-17), 33.3 (CH_3_-29), 34.0 (CH_2_-21), 34.4 (CH_2_-2), 36.3 (C-10), 37.0 (CH_2_-22), 39.0 (C-14), 40.6 (CH_2_-1), 41.6 (CH_2_-7), 42.5 (C-8), 46.7 (CH_2_-19), 47.3 (CH-9, 18), 48.7 (C-4), 56.5 (CH-5), 69.3 (CH-6), 121.2 (CH-12), 144.5 (C-13), 216.6 (C-3), ppm; HRMS (ESI) *m*/*z*: calcd. for C_30_H_48_O_2_ [M]^+440.3710^ found 440.3700. The spectroscopic data were compared to the literature and found identical with literature reported data [21, 22]; furthermore, the chemical structure of daturaolone (**1**) was confirmed by X-ray crystallography data (Figure 1).

The crude methanolic extract and various fractions and also daturaolone (**1**) were screened for *α*-glucosidase inhibitory activity; the tested extracts, fraction, and isolated daturaolone (**1)** were found active on *α*-glucosidase.

Diabetes mellitus is a dominant disease in developed as well as developing countries; it is a prominent metabolic turmoil or in other words an abnormal postprandial increment of blood glucose level. The controller of postprandial hyperglycemia is accepted to be imperative in the cure of diabetes mellitus. *α*-Glucosidase produced from the intestinal chorionic epithelium is responsible for the degradation of the glucose molecules. The inhibitors of *α*-glucosidase (EC 3.2.1.20) are potent antidiabetic agents. Several *α*-glucosidase inhibitors including voglibose and acarbose obtained from a natural source can sufficiently control blood glucose levels and have been used clinically in the cure of diabetes mellitus. Among several natural products, only a few *α*-glucosidase inhibitors are industrially available [22–24].

The obtained results indicated that chloroform fraction exhibited maximum inhibitory potential 90.8% with IC_50_ value 160.2 ± 1.85 *μ*M followed by ethyl acetate fraction 82.7% with IC_50_ of 86.2 ± 2.00. The methanolic extract also showed promising activity with % inhibition of 80.2% with IC_50_ value 110.6 ± 2.66 *μ*g, respectively. The daturaolone isolated from most active frication showed potent activity 98.7% with IC_50_ value 830.4 ± 2.01 *μ*M as compared to the standard drug having 90.2% inhibition with IC_50_ value 840.4 ± 1.74 *μ*M (Table 1). The bioactivity of the chloroform extract are correlated due to the presence of daturaolone.

*β*-Secretase processing of amyloid precursor protein (*β*-APP) is the 1^st^ step in a pathway leading to the formation of A*β* (amyloid); therefore, it is a major target for the development of a drug for the cure of Alzheimer's disease (AD). The *β*-secretase enzyme (BACE1) is important for the production of ab. It has been documented that BACE1 level is high in Alzheimer's disease (AD); therefore, the study BACE1 inhibition is helpful in developing new Alzheimer's disease (AD) therapies [25]. Keeping the significance of BACE1 activity in mind, the crude methanolic extract and various fractions and daturaolone (**1**) were screened *β*-secretase inhibitory potency. Among tested fractions, the chloroform fractions showed excellent activity against *β*-secretes (BACE1) enzyme with % inhibition 88.27% with IC_50_ value of 304.21 ± 2.98 *μ*g followed by methanolic extract 78.42% with IC_50_246.51 ± 1.98 *μ*g, respectively. The ethyl acetate fractions also showed good inhibition with 67.98% activity with IC_50_ value 87.93 ± 2.87 *μ*M, while *n*-hexane fraction was found the least active (Table 2). The daturaolone (**1**) was found significantly active against BACE1 enzyme with % inhibition 95.19 and IC_50_ value 260.70 ± 1.87 *μ*M as compared to standard having % activity 94.21 and IC_50_ value 289.24 ± 1.60 *μ*M.

The bioactivity of daturaolone (**1**) toward *α*-glucosidase and *β*-secretase suggests that the bioactivity is due to the keto moiety at position C-3 and a hydroxyl group at position C-6 which may bind with residues in the active sites of selected enzymes.

## 5. Conclusion

Phytochemicals can provide an excellent pharmacophore template for novel drug discovery used for the cure of various diseases. The crude extract/fraction and daturaolone (**1**) exhibited excellently *α*-glucosidase inhibition activity. Similarly, the extract/fractions and isolated daturaolone (**1**) showed potent and *β*-secretase activity. In this study, the extracts and natural daturaolone (**1**) inhibit *α*-glucosidase and *β*-secretase significantly and can be used as an excellent template compound for the new drug used in diabetes and AD.

## Figures and Tables

**Table 1:** *α*-Glucosidase inhibitory activity of extract/fractions and isolated daturaolone (**1**).

Sample/standard	Concentration	% inhibition	IC_50_ ± SEM (*μ*M)
Hexane	0.2 *μ*g	42.6	NA
Chloroform	0.2 *μ*g	90.8	160.2 ± 1.85
Ethyl acetate	0.2 *μ*g	82.7	86.2 ± 2.00
Methanol	0.2 *μ*g	80.2	110.6 ± 2.66
Daturaolone (**1**)	0.2 *μ*M	98.7	830.4 ± 2.01
Standard	0.2 *μ*M	90.2	840.4 ± 1.74

IC_50_: minimum inhibitory concentration; SEM: standard error of mean; *μ*M: micromolar; Standard: acarbose.

**Table 2:** *β*-Secretase inhibitory activity of extract/fractions and isolated daturaolone (**1**).

Sample/standard	Concentration	% inhibition	IC_50_ ± SEM (*μ*M)
Hexane	0.2 *μ*g	38.28	NA
Chloroform	0.2 *μ*g	88.27	304.21 ± 2.98
Ethyl acetate	0.2 *μ*g	67.98	87.93 ± 2.87
Methanol	0.2 *μ*g	78.42	246.51 ± 1.98
Daturaolone (**1**)	0.2 *μ*M	95.19	260.70 ± 1.87
Standard	0.2 *μ*M	94.21	289.24 ± 1.60

IC_50_: minimum inhibitory concentration; SEM: standard error of mean; *μ*M: micromolar; Standard: (Ans670, Sta671, Val672)-amyloid-*β*/A4 precursor protein 770 fragments 662-675).

## Data Availability

The data such as spectra and the associated analysis used to support the investigation of this study are available from corresponding authors upon request.

## Supplementary Materials

Supplementary Materials Figure 1: ^1^H-NMR NMR spectra of daturaolone (1) isolated from Datura metel. Figure 2: ^13^C-HNMR spectra of daturaolone (1) isolated from Datura metel. Figure 3: 2D H-NMR spectra of daturaolone (1) isolated from Datura metel. Figure 4: EI-MS spectra of daturaolone (1) isolated from Datura metel. Figure 5: FT-IR spectra of daturaolone (1) isolated from Datura metel.
