# Supplementary material for: Potent In Vitro α-Glucosidase and β-Secretase Inhibition of Amyrin-Type Triterpenoid Isolated from Datura metel Linnaeus (Angel's Trumpet) Fruits
Source: Biomed Res Int. 2020 Aug 25;2020:8530165. doi: 10.1155/2020/8530165 (PMC7468596; doi:10.1155/2020/8530165)
Supplement: Supplementary Materials — Figure 1: 1H-NMR NMR spectra of daturaolone (1) isolated from Datura metel. Figure 2: 13C-HNMR spectra of daturaolone (1) isolated from Datura metel. Figure 3: 2D H-NMR spectra of daturaolone (1) isolated from Datura metel. Figure 4: EI-MS spectra of daturaolone (1) isolated from Datura metel. Figure 5: FT-IR spectra of daturaolone (1) isolated from Datura metel. [file 8530165.f1.docx]

**Supplementary file**

**Potent *in vitro* *α*-Glucosidase and *β*-secretase inhibition of amyrin type triterpenoid isolated from *Datura metel* Linnaeus (Angel’s trumpet) fruits**

**Saud Bawazeer^1,^ Abdur Rauf^2^, Sami Bawazeer^3^**

^1^Department of Pharmaceutical Chemistry, Faculty of Pharmacy, Umm Al-Qura University, Makkah, P.O. Box 42, Saudi Arabia

^2^Department of Chemistry, University of Swabi, Swabi-Anbar, KPK, Pakistan

^3^Department of Pharmacognosy, Faculty of Pharmacy, Umm Al-Qura University, Makkah, P.O. Box 42, Saudi Arabia

*Correspondence should be addressed to Abdur Rauf; [mashaljcs@yahoo.com](mailto:mashaljcs@yahoo.com), Saud Bawazeer; [ssbawazeer@uqu.edu.sa](mailto:ssbawazeer@uqu.edu.sa)


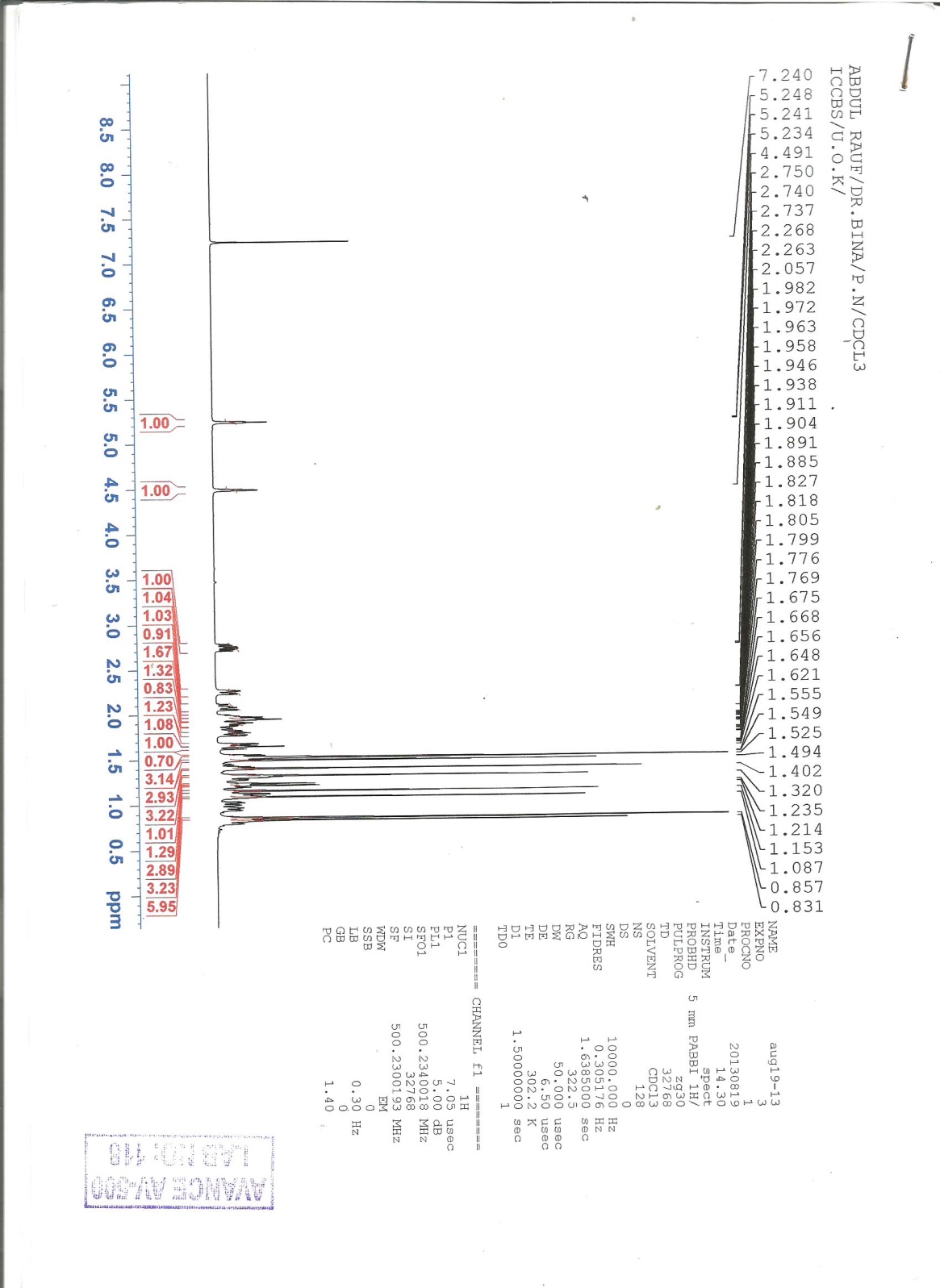


**Figure 1:** ^1^H-NMR NMR spectra of daturaolone (**1**) isolated from *Datura metel*


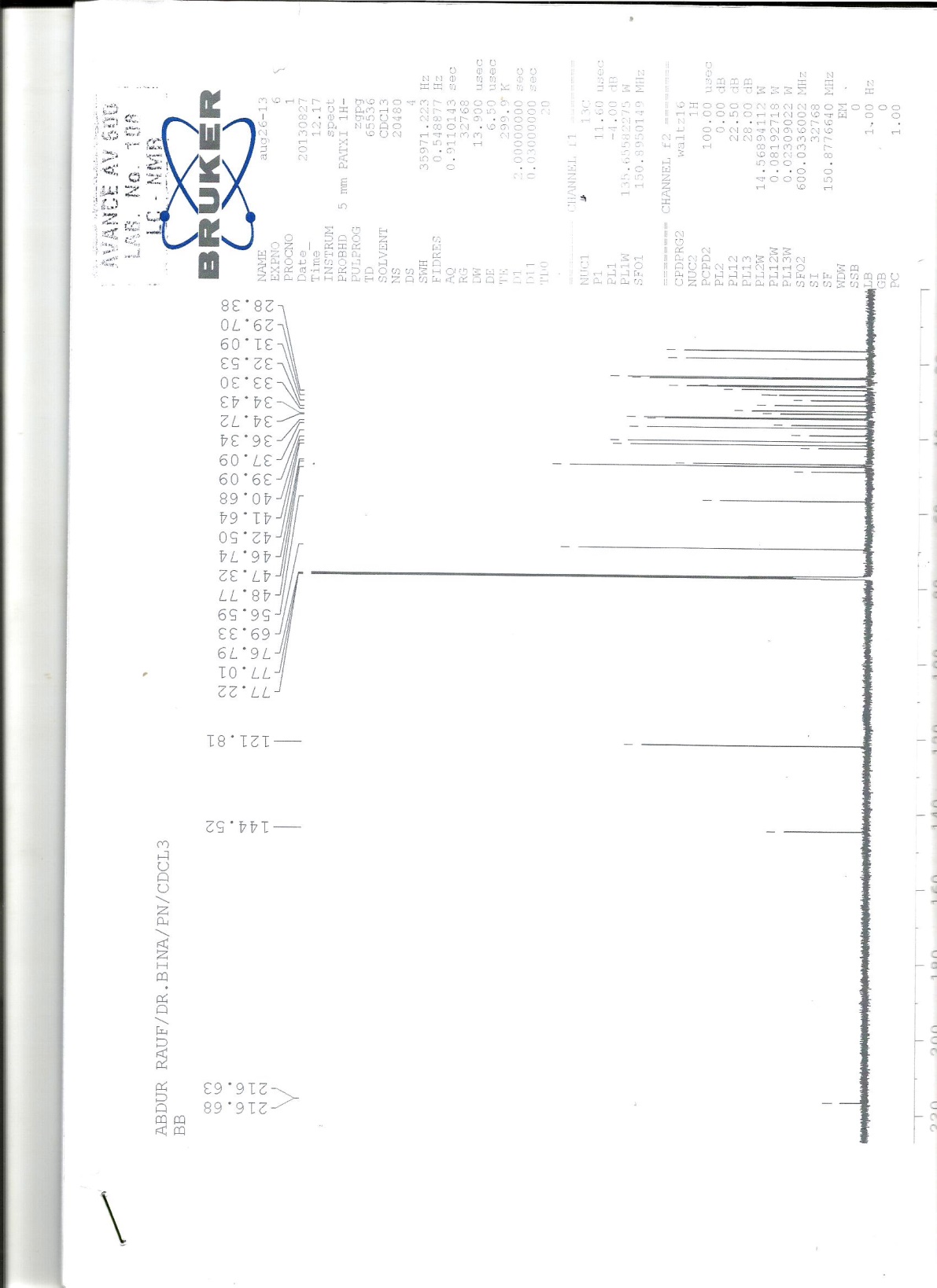


**Figure 2:** C^13^-HNMR spectra of daturaolone (**1**) isolated from *Datura metel*


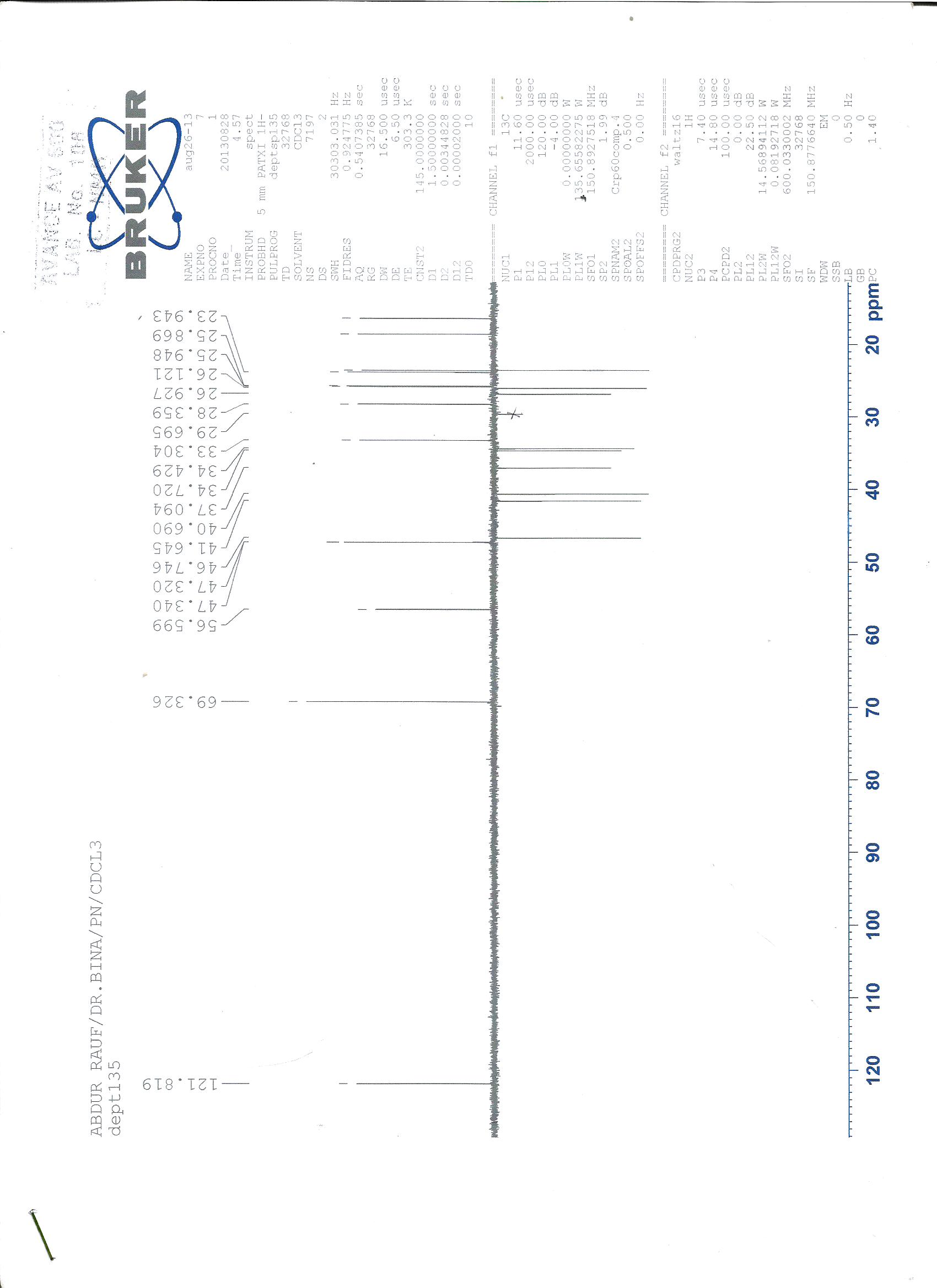


**Figure 3:** 2D H-NMR spectra of daturaolone (**1**) isolated from *Datura metel*


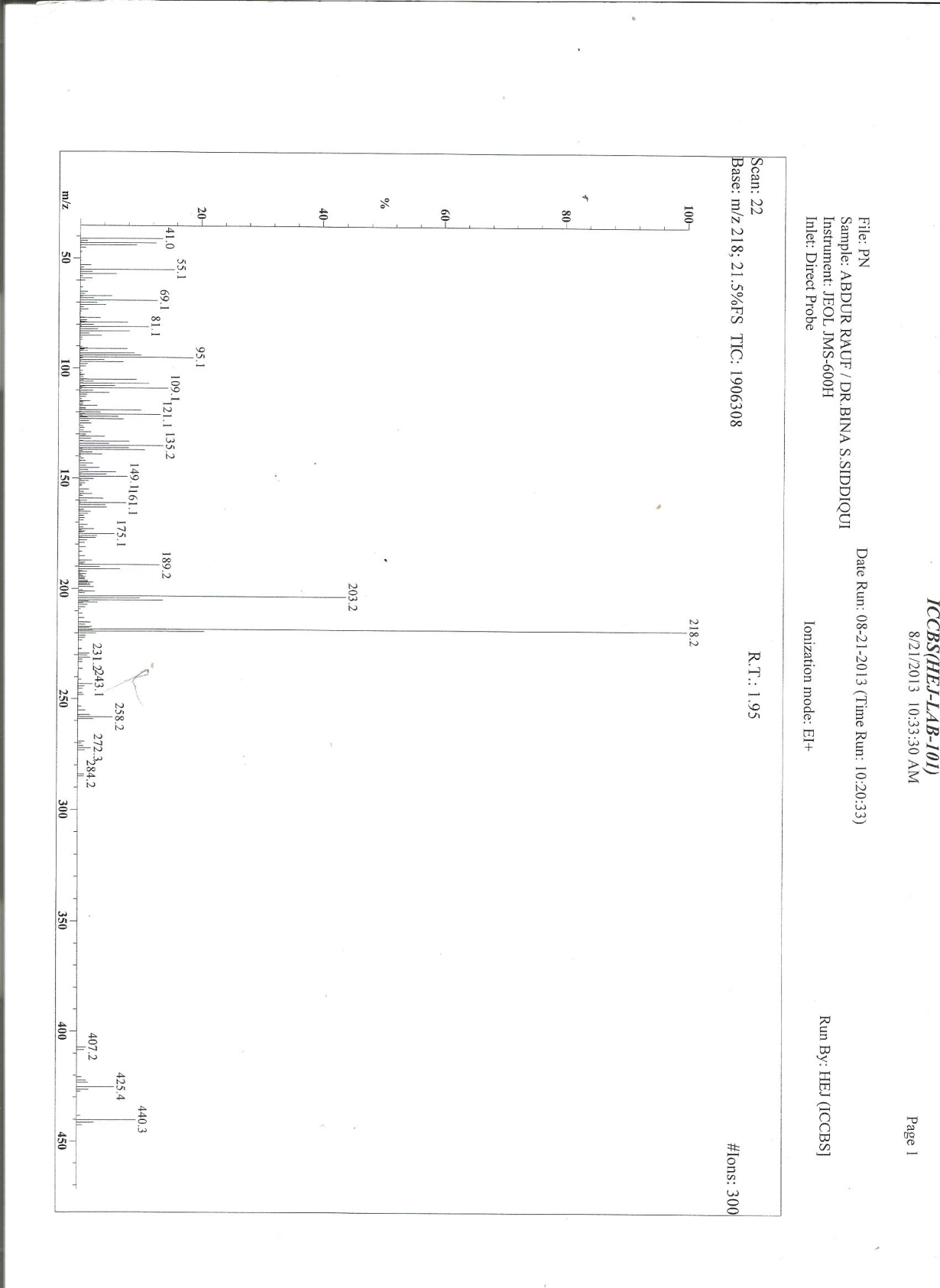


**Figure 4:** EI-MS spectra of daturaolone (**1**) isolated from *Datura metel*


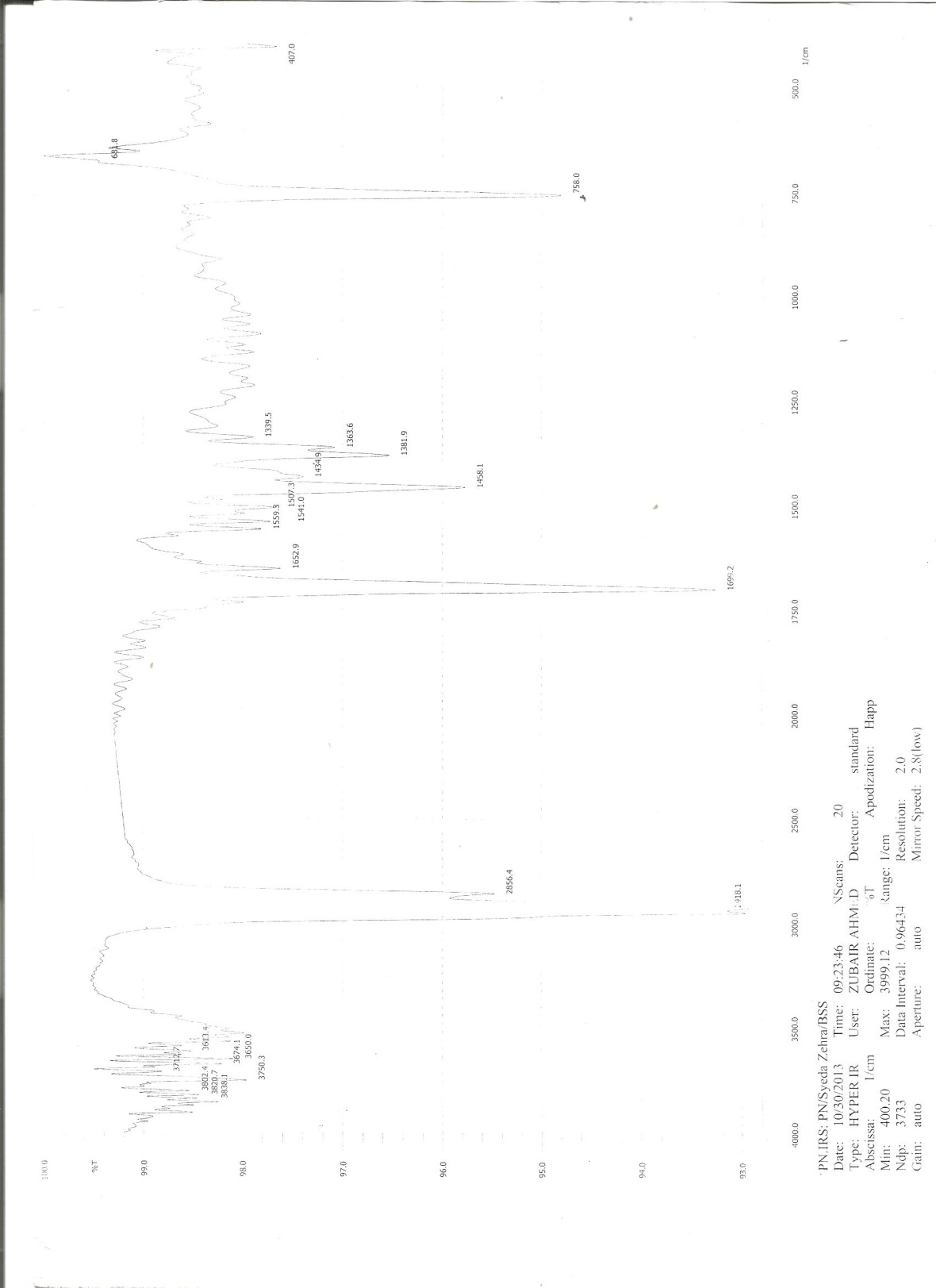


**Figure 5:** FT-IR spectra of daturaolone (**1**) isolated from *Datura metel*
